# Semi-Automated Biomarker Discovery from Pharmacodynamic Effects on EEG in ADHD Rodent Models

**DOI:** 10.1038/s41598-018-23450-y

**Published:** 2018-03-26

**Authors:** Tatsuya Yokota, Zbigniew R. Struzik, Peter Jurica, Masahito Horiuchi, Shuichi Hiroyama, Junhua Li, Yuji Takahara, Koichi Ogawa, Kohei Nishitomi, Minoru Hasegawa, Andrzej Cichocki

**Affiliations:** 1 RIKEN Brain Science Institute, Hirosawa, Wako, Saitama, Japan; 2 SHIONOGI & Co., Ltd., Futaba, Toyonaka, Osaka, Japan

## Abstract

We propose a novel semi-automatic approach to design biomarkers for capturing pharmacodynamic effects induced by pharmacological agents on the spectral power of electroencephalography (EEG) recordings. We apply this methodology to investigate the pharmacodynamic effects of methylphenidate (MPH) and atomoxetine (ATX) on attention deficit/hyperactivity disorder (ADHD), using rodent models. We inject the two agents into the spontaneously hypertensive rat (SHR) model of ADHD, the Wistar-Kyoto rat (WKY), and the Wistar rat (WIS), and record their EEG patterns. To assess individual EEG patterns quantitatively, we use an integrated methodological approach, which consists of calculating the mean, slope and intercept parameters of temporal records of EEG spectral power using a smoothing filter, outlier truncation, and linear regression. We apply Fisher discriminant analysis (FDA) to identify dominant discriminants to be heuristically consolidated into several new composite biomarkers. Results of the analysis of variance (ANOVA) and t-test show benefits in pharmacodynamic parameters, especially the slope parameter. Composite biomarker evaluation confirms their validity for genetic model stratification and the effects of the pharmacological agents used. The methodology proposed is of generic use as an approach to investigating thoroughly the dynamics of the EEG spectral power.

## Introduction

Neurogenic cognitive and behavioural disorders constitute an ever-growing challenge to societies^1^, barely met by the continuing development of pharmacological treatments. Biomarker discovery is essential to drug development, and also constitutes a formidable challenge in evaluating the effects of newly developed pharmacological agents. In particular, non-invasive biomarkers are of high value in diagnosing diseases and evaluating disease progression and the efficacy of medication. This applies specifically to diseases of pathoneurological origin, where the cognitive and behavioural health of the individual is affected. The existence of a battery of non-invasive biomarkers capable of identifying neurogenic alteration of normal functioning and capturing the response to pharmacological agents would be of great importance in clinical practice. Specifically, electroencephalography (EEG)-based markers have the potential of serving as such a battery, due their noninvasiveness and well-established recognition in clinical practice.

Indeed, recent evidence indicates that quantitative electroencephalogram (QEEG) is a powerful tool in pharmaco-EEG applications. The identification of treatment responsive QEEG subtypes has been described in depression^2,3^, obsessive compulsive disorder^4,5^ and schizophrenia^6^, suggesting that understanding the underlying neurophysiology of the patient can contribute significantly to treatment optimization. QEEG has been shown to distinguish between attention deficit/hyperactivity disorder (ADHD) responders and non-responders to stimulant medication^7–9^.

Unfortunately, to date there is no established methodological approach to the design of QEEG derived biomarkers, in particular, in an automatic, objective and reproducible way. Also, the methodological approaches currently used and reported underutilise the informational contents of EEG signals. Neither do they provide a consistent roadmap to obtain novel combinations of information-bearing components of EEG signals. Here, we propose a methodology aimed at resolving this, and we design a sequence of generic steps converging towards obtaining novel EEG markers in neuropathological disorders. We illustrate our methodology using a specific experiment, involving three genetic strains of rat, namely spontaneously hypertensive rat (SHR), Wistar-Kyoto rat (WKY), and Wistar rat (WIS), with the goal of reproducing leading behavioural characteristics of these strains, namely various degrees of locomotor activity and impulsivity.

SHR is a well-characterised and fairly readily available animal model of ADHD, widely used for pharmacological studies on the effects of ADHD treatments. A number of characteristics of SHR have been reported, such as inattention^10^, impulsively^11^, hyperactivity^12,13^, working memory impairment^14^, dopaminergic dysfunctions^15^, and genetics^16^. The SHR model was developed in the 1960s^17^ by inbreeding the WKY, which exhibited high systolic blood pressure. Therefore, SHR develops hypertension over time^18^ and as a result, SHR develops a significant and unexpected hyperactivity when compared with WKY rats^19,20^. In addition to hyperactivity, SHR displays inattention and distinct impulsivity which can, however, be alleviated by methylphenidate (MPH), amphetamine and other drugs involved in the treatment of ADHD^21,22^. Together, these similarities support the validity of SHR as animal model of ADHD. Somkuwar *et al*. studied the effects of ADHD treatments on cocaine self-administration by using SHR, WKY, and WIS rats^23,24^, where WKY served as an animal model of a depression patient^25^, WIS served as an animal model of a normal control group, and both were compared with SHR.

These strains have been extensively studied, primarily by Sagvolden *et al*.^10,26–28^. It is, however, evident that SHR as an animal model of ADHD is not unambiguous. For example, amphetamine and MPH, which reduce hyperactivity in ADHD children, induce an increase in activity in both SHR and WKY rats^29,30^. The extent of the stimulation has been found to be lower in SHR but it is in stark contrast to clinical studies^31^. In the open-field test, the rat is exposed to novel and unnatural surroundings and its behavioural reactions are to an important degree determined by this stress. SHR displays a marked increase in exploratory rearing activity in this test^26,30^. Brain dopamine systems play an important role in open-field locomotor activity and exploratory behaviour. These findings support the essential role of dopamine in the development of spontaneous hypertension in rats^30^.

We test our methodology on two types of ADHD pharmacological agents/medications, namely methylphenidate (MPH), a stimulant acting through inhibition of catecholamine reuptake, primarily as a dopamine reuptake inhibitor, and a selective inhibitor of norepinephrine transporters (SNRI), atomoxetine (ATX). In an animal study, Koda *et al*.^32^ analysed the effects of acute and chronic administration of both MPH and ATX on mice and found selective activation of the prefrontal catecholamine systems. Umehara *et al*.^33^ analysed the effects of ATX and MPH on locomotion and prefrontal monoamine release in SHR rats. They found that both ATX and MPH increased the extracellular levels of norepirephrine (NE) and dopamine (DA) in the prefrontal cortex (PFC) of SHR. Urban *et al*.^34^ showed that the juvenile prefrontal cortex is supersensitive to MPH, due to significant depressive effects on pyramidal neurons of both single dose and chronic treatment with MPH at low doses (1 mg/kg). However, the same dose in adult rats resulted in excitatory effects. Administration of stimulants such as MPX appears to correct prefrontal hypoactivity which is considered to be the leading cause of ADHD. In the juvenile PFC, even a dose of MPH thought to be within the clinically relevant range of 1 mg/kg may in fact cause excessively high levels of DA and NE. The majority of studies investigating ATX have assumed noradrenaline to be primarily affected, even though ATX can target prefrontal dopamine at only slightly higher doses. In conclusion, the majority of studies involving ATX to date appear not to have employed an adequate animal model of ADHD or, as some researchers point out^35^ have used administration methods incongruous with human use, where the drug is administered in tablet form.

ADHD is a developmental disorder affecting children, in particular, which leads to attention deficit, impulsiveness and hyperactivity. Its ethiology is, however, not fully understood^36^. ADHD is caused by multiple genetic and environmental factors and is thought to be related to an imbalance in the functioning of neural systems in the brain. In children who are diagnosed as having ADHD, the functions of DA and NE transporters are overly working, while the neurotransmission function is lagging. This systemic imbalance is considered to cause symptoms of attention deficit, impulsiveness, and hyperactivity^37,38^. Decreased function of working memory caused by the destabilized neurotransmission is also related to the attention deficit in ADHD^39^. Further, ADHD is likely to occur in conjunction with rebellious behavioural disorder, depression, anxiety disorder and tic disorder^40,41^. The exact causes and mechanisms of ADHD development remain unknown, leaving precaution, diagnosis, and treatment as open, unsolved problems. MPH^42^ and ATX^43^ are widely used in symptomatic treatment of ADHD.

QEEG has been shown to have sensitivity and specificity levels varying from 90% to 98% in discriminating normal subjects from those with ADHD and ADHD children from children with learning difficulties^8,44–46^. QEEG has also proved useful in the management of treatment response to stimulant medication. A number of studies have investigated changes in the EEG due to stimulant medications, with the majority of studies finding that the stimulants result in some normalization of the EEG. Swartwood *et al*.^47^ and Lubar *et al*.^48^ failed to find changes in EEG power due to stimulant medication, but Chabot *et al*.^49^ found that 56.9% of a group of children with ADHD showed normalization of the EEG after the administration of a stimulant. Loo *et al*.^50^ found that after the administration of methylphenidate, good responders had decreased theta and alpha but increased beta activity in the frontal regions, while poor responders showed the opposite EEG changes: Skirrow *et al*.^51^ concluded normalisation of theta activity indicative of a role for dynamic impairments rather than stable cognitive deficits in cognitive performance and functional brain changes that are sensitive to administered task conditions. Clarke *et al*.^52–54^ consistently found that stimulant medications resulted in normalization of the EEG with a reduction in theta activity and an increase in beta activity.

Barry *et al*.^55^ investigated the effects of a single dose of ATX on the electroencephalogram (EEG) and performance of children with ADHD. They concluded that ATX can produce substantial normalization of the ADHD QEEG profile, together with behavioural performance improvements. It has been previously shown that ATX increased extracellular concentrations of NE and DA in the PFC^56^. Furthermore, chronic administration of ATX for 21 days also increased NA and DA levels in the prefrontal cortex^32^. Leuchter *et al*.^57^ used the theta cordance index in predicting ATX treatment response in adult ADHD. Left temporo-parietal cordance in the theta frequency band after one week of treatment was associated with ADHD symptom improvement and quality of life measured at 12 weeks in ATX-treated subjects, but not in those treated with a placebo. There is only one study that investigated the acute treatment effect of 20 mg of ATX in children and adolescents with ADHD^55^. The EEG was recorded after 1 h of ATX administration. Acute ATX administration produced a significant decrease in posterior absolute theta and an increase in absolute beta (especially in right and midline anterior regions). Relative delta was increased, particularly in central regions, and relative beta was globally increased. There were no significant medication effects on absolute alpha activity. However, this study has minimal implications for the long-term effects of ATX on QEEG changes which were investigated by Chiarenza *et al*.^58^. They found increased absolute power in alpha and delta in frontal and temporal regions in the responders compared with widely distributed increased absolute power in all frequency bands in non-responders.

However, it should be noted that Robbins and Arnsten^59^ point out that an inverted U-shape function links the efficiency of behavioural performance to the level of activity in the ascending monoaminergic systems. A general principle that has emerged in the past decade in considering the functions of the chemical modulatory inputs to the PFC has been that of the Yerkes-Dodson inverted U-shaped function linking the efficiency of behavioural performance to the level of activity in the ascending monoaminergic (mainly DA- and NE-ergic) systems. The inverted U dose response has been demonstrated with pharmacological agents in both animals, e.g.^60,61^, and humans^62^. Further complications have related to heterogeneity of function within the PFC and the fact that the Yerkes-Dodson relationships may posit different U-shaped functions depending on the nature of the task; therefore, a level of monoaminegic function optimal for one may be sub- or supraoptimal for another. In particular, in contrast to the essential effects of moderate levels of catecholamines, very high levels of catecholamine release in the PFC during stress exposure markedly impair working memory function through network collapse and suppression of PFC cell firing. However, as already observed by Pliszka *et al*.^63^, it seems unlikely that ADHD is related to a simple hypo-functioning of the dopamine system, Indeed, the complex multistage hypothesis of ADHD suggested by Pliszka *et al*. remains a plausible model of the complex interactions involved in ADHD ethiology.

Since the symptoms of ADHD are caused by an anomaly in brain network function, characterised by an increase in slow-wave (delta and theta) characteristics in particular^64^, it is worth analyzing the EEG of ADHD patients or ADHD-like animals. EEG is, however, a small, distortion and artefact prone voltage signal measured on the scalp, reflecting electrical potential transmission of neuronal population activities in the brain. EEG forms high-dimensional digital data, and due to noise content and high complexity, it requires analysis using state-of-the-art sophisticated statistical and probabilistic methods of signal processing.

There is no universal agreement regarding the utility of EEG-based markers for ADHD. Literature reviews reveal individual EEG spectral component-based biomarkers, often reporting contradictory findings^65^. The lack of a methodological basis for discovering more robust biomarkers is evident in both clinical and animal laboratory experimental research, leading to difficulties in establishing translational markers and testing their validity. In this study, we conducted an experiment to investigate the differences in EEG spectral power with and without MPH and ATX administration to SHR, WKY and WIS genetic strains. This experiment was designed to quantify: i) the effects of these ADHD treatments on each particular genetic strain considered, ii) the difference between individual rodent models, and iii) the effects of the pharmacological agents used. To investigate the effects of pharmacological agents on the individual rodent models, we applied a novel approach to the semi-automatic biomarker design, which consists of three steps of feature extraction and of three types of statistical analysis. The feature extraction is an essential procedure to quantify the dynamical records of EEG spectral power into meaningful parameters. In general, temporal records of EEG spectral power form high dimensional data arrays. They also contain substantial levels of noise and outliers. For these reasons they are complicated to evaluate. We use a median filter to reduce noise, a functional boxplot algorithm to detect outliers, followed by linear regression to quantify the signals in terms of slope and intercept parameters. Linear regression has several advantages compared to simply calculating the average value. Firstly, linear regression significantly reduces information loss of time-varying EEG signals with only one additional parameter. Secondly, slope and intercept parameters contain potentially important time-varying information, in particular that of the pharmacodynamic effects of the agents. Since the evaluation of the slope and intercept parameters for the EEG pharmacodynamic effects is a novel approach, we consider this to be a major methodological contribution of this work.

For the statistical analysis, analysis of variance (ANOVA), t-test, and Fisher discriminant analysis (FDA) were applied. ANOVA evaluates the significance of extracted feature parameters: slope, intercept and temporal average of spectral power. The t-test finds the significant difference between individual treatments in each rodent model. The FDA reveals dominant biomarkers for stratification of the animal model and effects of pharmaceutics via EEG spectral power features, and evaluates dominant differences in parameters of individual EEG frequency bands before and after the administration of the agent. We used the FDA to discover biomarkers in a semi-automatic way.

We have organized the remainder of the paper as follows. Firstly, in the Materials and Methods section, we explain the experimental environment and introduce the proposed methodology entailing analysis methods for noise reduction, outlier detection, feature extraction (linear regression), statistical tests and classification. Next, we present and evaluate the results of the analysis in the Results section. Finally, in the Discussion section, we discuss the advantages of the novel methodology proposed.

## Materials and Methods

### Animals

SHR, WKY, and WIS rats were obtained from Charles River Laboratories Japan, Inc. at 5 weeks of age. There were 10 animals of each genetic strain; 30 animals were obtained in total. The animals were supplied with standard food and water ad libitum under controlled temperature and humidity with a 12/12 hours light/dark cycle. The animal study protocol for this study was carefully reviewed by the Institutional Animal Care and Use Committee (IACUC) and then approved at Shionogi & Co., Ltd. by the director of the institute, based on the report by the IACUC. All experiments were performed in accordance with relevant guidelines and regulations.

### Pharmacological Agents

MPH hydrochloride and ATX hydrochloride were obtained from Sigma-Aldrich and Tokyo Chemical Industry Co., Ltd., respectively. Both pharmacological agents were dissolved in a saline solution (0.9% NaCl) and were administered by intraperitoneal dosage. Three dose ratios of MPH (0.3, 1.0, and 3.0 [mg/kg]) and two dose ratios of ATX (1.0 and 2.0 [mg/kg]) were prepared.

### Experiment

**Figure 1:**
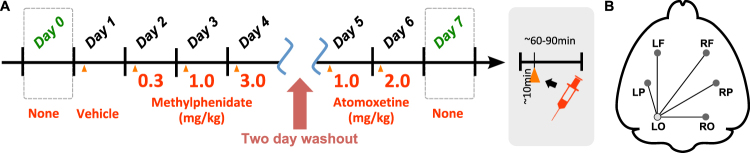
Experimental procedure and signal acquisition. (**A**) EEG signals of each animal were recorded 10 minutes before and 60–90 minutes after injection of medication. No medication was administered on days 0 and 7. Day 4 was followed by a two-day washout period. (**B**) Schematic layout of electrode locations, left occipital (LO) electrode was used as a reference.

The rats had electrodes implanted on their scalps at 5 weeks of age, then were allowed to recover for 5–7 days after electrode implantation. After the recovery from surgery, EEG recording was done at 6–7 weeks of age. Six EEG electrodes were implanted on the left and right frontal (±2.0 mm lateral and 3.2 mm anterior from the bregma), parietal (±3.5 mm lateral and 1.8 mm posterior from the bregma) and occipital cortex (±2.0 mm lateral and 5.2 mm posterior from the bregma) of the rodents’ scalps. The left occipital cortex electrode was used as a reference. With this set-up, five time series of EEG recordings were obtained. The time series of EEG signals were recorded for 1.0–1.5 hours each day according to the protocol devised (see Fig. 1). Eight experiment days were considered: no agent administration on Day 0, vehicle (saline) on Day 1, 0.3 [mg/kg] of MPH on Day 2, 1.0 [mg/kg] of MPH on Day 3, 3.0 [mg/kg] of MPH on Day 4, 1.0 [mg/kg] of ATX on Day 5, 2.0 [mg/kg] of ATX on Day 6, and no agent administration on Day 7. This provided a total of five time series of eight experimental conditions for each individual animal.

### Data Analysis

**Figure 2:**
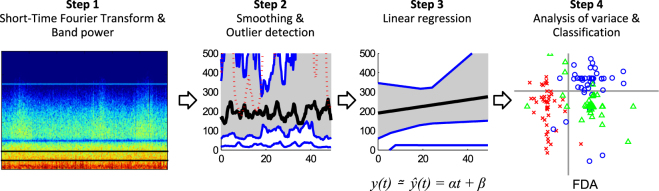
Schematic illustration of the data processing flow and the main data processing concepts utilised.

The main objective of this study was to investigate the effects of the pharmacological agents applied to each particular genetic strain of the rats used, as reflected in the EEG time series. However, time series recordings of extensive duration contain a range of brain activity modalities due to the particular behavioural characteristics of individual animals. This results in both a substantial inter-subject/animal variability and in intra-subject/animal variability in the signals observed. Furthermore, EEG signals commonly include noise, artefacts and outliers of various origins. Our purpose was to extract meaningful common dynamical behaviour due to individual pharmacological agents applied for each separate genetic strain of rat. To this end, we applied a dedicated sequence of pre-processing steps: smoothing filter, outlier detection, and linear regression followed by the analysis of the results using ANOVA, t-test and FDA. In Fig. 2, we schematically illustrate the flow of data processing and graphically show the main data analysis concepts involved.

#### Time-frequency analysis

We considered 9 frequency sub-bands of the EEG spectral power labelled as follows: Total (0–250 Hz), lDelta (low Delta in 0.1–1.5 Hz), Delta (1–4 Hz), Theta (4–8 Hz), Alpha (8–12 Hz), Beta (12–30 Hz), Gamma (30–55 Hz), High (70–170 Hz), and vHigh (very high frequencies in 190–250 Hz). Frequency band-specific dynamical behaviour of the spectral power of all the individual sub-bands was computed for all the time series for the entire duration of each recording session at 250 ms steps. These temporal records of spectral sub-band power time series were cropped at 1-hour duration, records of days were aligned so that the time of injection fell in the sample occurring 9 minutes after the first sample. The resulting dataset used for the analysis was stored as a high-dimensional array consisting of values of spectral power for 9 frequency sub-bands, 14396 time samples, 5 EEG channels, 8 experimental days, 10 animals, and 3 genetic strains.

#### Smoothing via median filter

The recorded EEG signals included a range of unavoidable noise caused by peripheral devices, external sounds and animal motor activity. Since the focus of this study was on extracting the dynamical information contained in the temporal records of EEG spectral power, such persistent noise, transient nonstationarities, bursts and isolated spikes were undesirable. In order to reduce the degenerative effect of these noise phenomena on signal quality, we applied a median filter. For the details of the median filter, see Supplementary Note 1 and Supplementary Figure 1.

#### Outlier detection via functional boxplot

After the median smoothing, signals from individual animals of the same genetic strain still possessed high inter-individual variability. As the next pre-processing step, we identified the central region of the group of signals and detected possible outlying signals. A total of 216 groups consisting of 3 genetic strains, 8 experimental days, and 9 frequency sub-bands were considered at this stage. In each group, there were 50 signals consisting of 5 recording channels and 10 animals in each genetic strain and each frequency sub-band. In order to find the outliers in a group, we employed the technique of the functional boxplot^66^, which is a generalisation of the method of the boxplot for the group of scalar values, suitable for application to the group of time-varying continuous signals considered here. For the details of the functional boxplot, see Supplementary Note 2 and Supplementary Figures 2, 3.

#### Linear regression and mPower

**Figure 3:**
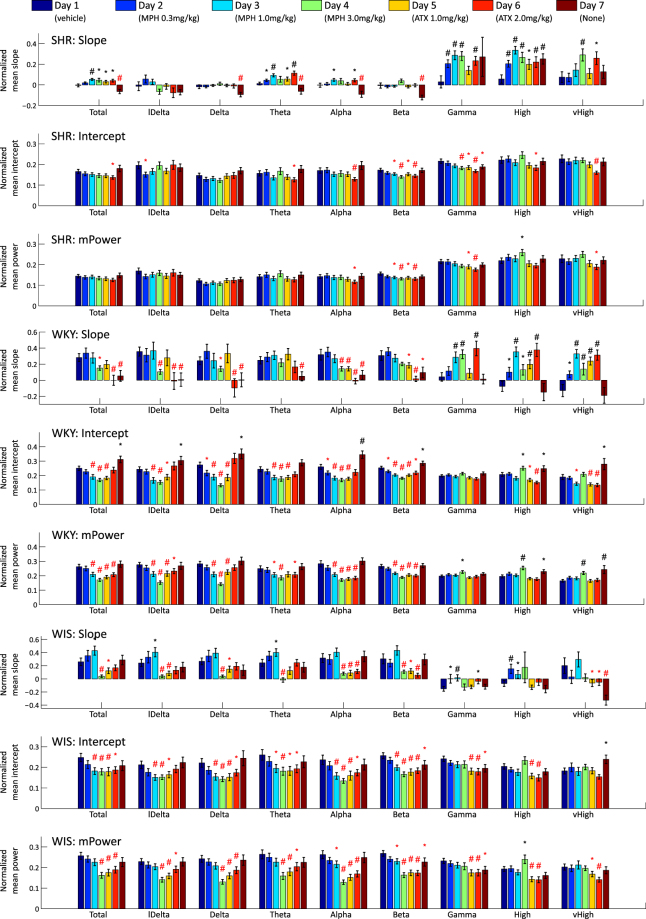
Result of experimental conditions in SHR, WKY, and WIS: Average and standard error of the mean (SEM) of slope, intercept and mPower parameters are depicted as bar and error bar. The marks ‘*’ and ‘#’ indicate the results of the statistical significance test, the one-tailed t-test, between the vehicle and other specific pharmacological agent administration for which the significant levels are 0.05 and 0.01, respectively. Positive and negative significance are colour-coded using black and red, respectively.

To date, in almost all EEG studies of both rodent and human ADHD, EEG spectral power has been assessed using mean values of the total spectral power. Such mean values are usually obtained using averaging of spectral power, to obtain one grand mean value per frequency sub-band (mPower). This is performed using short-time binning of each frequency band, which are in turn summed up as follows:

$$\frac{1}{N}{\sum }_{n=1}^{N}\,y({t}_{n}),$$

where *y*(*t*) is the EEG spectral power per time bin *t*_*n*_ and $n=\mathrm{1,}\,\mathrm{...,}\,N$ is a running time index. This operation destroys all the temporal information contained in the original EEG. In Fig. 3 we present the results of the standard mPower for all the EEG frequency bands for all the days of our experiment and for all the animal strains investigated.

The crux of our novel approach here is in that we preserve the temporal dynamics of the EEG by retaining the values of the band-specific spectral power as a time-varying function *y*(*t*), where *y*(*t*) is the EEG spectral power per time bin *t*_*n*_ and *n* = 1, …, *N* is a running time index. Furthermore, we explicitly modelled this dynamics by using a parsimonious linear regression model.

The linear model is given by

1 $$y(t)\simeq  \sim \widehat{y}(t)=\alpha t+\beta ,$$

where *α* and *β* are the slope and intercept parameters. The appropriate values of *α* and *β* were obtained as

2 $$(\widehat{\alpha },\widehat{\beta })=\mathop{{\rm{argmin}}}\limits_{\alpha ,\beta }\sum _{t}{(y(t)-\alpha t-\beta )}^{2},$$

where *t* was aligned to the moment of the injection of the pharmacological agent at *t* = 0. The fitting was done over an interval 49 minutes long, starting 2 minutes after the injection of the pharmaceutical at the time annotated as *t* = 0. We removed from the analysis the first two minutes after injection, when the animal’s behaviour was particularly prone to impulsive responses to the human intervention.

The estimated value of the slope parameter $\widehat{\alpha }$ captures the dominant trend of the EEG power in the spectral sub-band considered. This is strongly related to the pharmacodynamical response of the animal of the particular genetic strain considered to the pharmacological agent used. The value of the intercept $\widehat{\beta }$ estimate reflects the estimated instantaneous response immediately after the intervention (injection), again in terms of the spectral sub-band of EEG considered for the particular genetic strain and in response to the pharmacological agent applied.

Both parameters were estimated for all spectral sub-bands, experiment days, and genetic strains considered and shown in Fig. 3. The results of the linear regression and mPower are shown in online Supplementary Figure 4.

#### Significance test

ANOVA and t-test: ANOVA of two-way design was conducted for the slope, the intercept, and the mPower parameters obtained for all the nine spectral sub-bands, while the experimental condition (agent administration type) and genetic strains were considered as factors. ANOVA evaluates the rate of between-class variance and within-class variance of some parameter. When between-class variance is large and within-class variance is small, the f-value becomes large, and its significance with respect to the parameter is evaluated based on the f-value. By using ANOVA, we evaluated the significance of the strain factor (three classes), experimental condition factor (eight classes), and their interaction with respect to each parameter involved.

Next, the t-test between individual experimental conditions was conducted for the parameters of slope, intercept, and mPower. The t-test evaluates the significance of the difference between two distributions based on their mean value, variance and the number of samples. We evaluated the effect of the experimental conditions on the three parameters for all the spectral frequency sub-bands and for all the rodent models/genetic strains considered.

#### Classification test

Fisher discriminant analysis: FDA is a feature extraction method to separate labelled samples into two classes. We applied the FDA to the extracted parameters of the three genetic strains (SHR, WKY, and WIS). There were a total of 150 samples, consisting of 5 EEG channels for 10 animals of each strain, where each sample was a 144-dimensional vector. These 144 dimensions consisted of 8 (experimental days) × 9 (spectral sub-bands) × 2 (parameters of slope and intercept).

A linear feature extraction model is given by

3 $${z}_{i}=\langle {\boldsymbol{w}},{{\boldsymbol{f}}}_{i}\rangle -{z}_{0},$$

where ***f***_*i*_ is the original data, z_*i*_ are the extracted features, ***w*** is a weighting parameter vector, and *z*_0_ is a threshold parameter. FDA is considered to be a method to infer ***w*** by using the following criterion:

4 $$\mathop{{\rm{\max }}}\limits_{w}\,\frac{{{\boldsymbol{w}}}^{T}{{\boldsymbol{S}}}_{B}{\boldsymbol{w}}}{{{\boldsymbol{w}}}^{T}{{\boldsymbol{S}}}_{W}{\boldsymbol{w}}},$$

where ${{\boldsymbol{S}}}_{B}\,:=({{\boldsymbol{\mu }}}_{1}-{{\boldsymbol{\mu }}}_{2})({{\boldsymbol{\mu }}}_{1}-{{\boldsymbol{\mu }}}_{2}{)}^{T}$ and ${{\boldsymbol{S}}}_{W}\,:={{\boldsymbol{S}}}_{1}+{{\boldsymbol{S}}}_{2}$ are between and within variance matrices, respectively, and ${{\boldsymbol{\mu }}}_{c}$ and ***S***_c_ for *c* ∈ {1, 2} are the mean vector and the covariance matrix of each class. The optimal weights can be analytically obtained as

5 $$\widehat{{\boldsymbol{w}}}={{\boldsymbol{S}}}_{W}^{-1}({{\boldsymbol{\mu }}}_{1}-{{\boldsymbol{\mu }}}_{2}\mathrm{)}.$$

There are several methods to estimate the threshold *z*_0_. For example, it can be obtained as

6 $${\widehat{z}}_{0}=\frac{{s}_{2}\langle \widehat{{\boldsymbol{w}}},{{\boldsymbol{\mu }}}_{1}\rangle +{s}_{1}\langle \widehat{{\boldsymbol{w}}},{{\boldsymbol{\mu }}}_{2}\rangle }{{s}_{1}+{s}_{2}},$$

where ${s}_{c}\,:=\sqrt{\langle \widehat{w},{{\boldsymbol{S}}}_{c}\widehat{w}\rangle }$. Each new sample ***f***_*new*_ was assigned to a particular class using the threshold and the weights obtained for the learning set

7 $${{\boldsymbol{f}}}_{new}\in \{\begin{array}{cc}({\rm{c}}{\rm{l}}{\rm{a}}{\rm{s}}{\rm{s}}1) & \langle {\boldsymbol{w}},{{\boldsymbol{f}}}_{new}\rangle \ge {z}_{0}\\ ({\rm{c}}{\rm{l}}{\rm{a}}{\rm{s}}{\rm{s}}2) & \langle {\boldsymbol{w}},{{\boldsymbol{f}}}_{new}\rangle  < {z}_{0}\,.\end{array}$$

We calculated the classification ratio via 10-fold cross-validation (CV) by using the FDA classifier for several variations of the feature vectors. In the 10-fold CV, we separated all 150 samples into 10 subsets, randomly and uniformly, obtained the classifier by using 9 subsets, and calculated the classification rate by using the remaining subset. We reiterated this classification procedure 10 times, using all possible combinations.

Semi-automatic biomarker search using FDA: We propose a new semi-automatic biomarker identification methodology, which consists of three steps: (1) detection of the best candidate spectral sub-bands, (2) obtaining the weighting parameters using FDA, and (3) spectral sub-band consolidation into the composite biomarkers.

**Figure 4:**
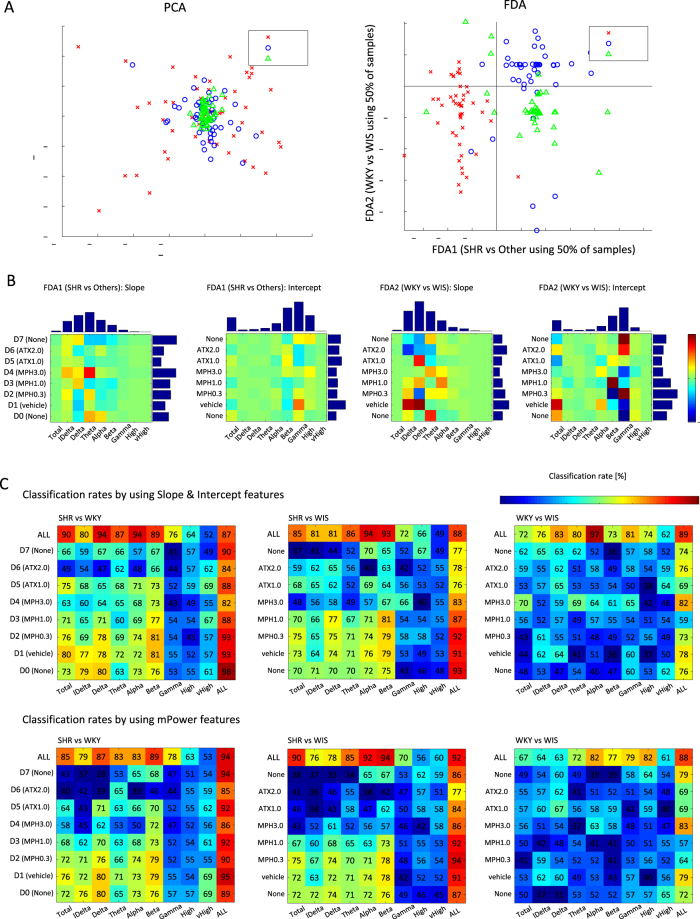
Results of FDA: (**A**) shows the visualization of all samples via PCA and FDA from a 144-dimensional original feature space, which consists of slope and intercept parameters for 9 frequency bands and 8 experimental condition days; (**B**) shows the visualization of weighting parameter obtained by FDA for two classification objectives. Individual bar plots depict the horizontal or vertical sums of the absolute values of classification outcomes; (**C**) shows the matrices of rounded averages of classification rates via 10-fold CV for all combinations of experimental conditions and sub-bands in each classification objective.

In the first step, we narrow down the spectral contents to the most relevant candidate sub-bands, using ANOVA and FDA. For example, the spectral bands of lower frequency (lDelta–Beta) appear to be more relevant than the higher frequency sub-bands (Gamma–vHigh), according to Table 1 and Fig. 4 (see Section “Results” for a detailed explanation). For this reason, we selected the low Delta–Beta sub-bands as candidates for the biomarkers.

**Table 1:** Results of two-way ANOVA analysis: each f-value and its significance is described.

bands	Slope			Intercept			mPower		
	Exp. Cond.	Strain	Interaction	Exp. Cond.	Strain	Interaction	Exp. Cond.	Strain	Interaction
Total	6.64**	42.90**	3.15**	10.80**	45.24 **	2.54 **	12.81 **	90.13 **	2.39**
lDelta	7.00 **	28.43 **	1.97 *	8.28**	20.19 **	3.68 **	9.19 **	57.82 **	3.44**
Delta	5.66 **	29.24 **	2.37 **	13.74**	56.21 **	3.44 **	15.98 **	130.68 **	3.29**
Theta	6.02 **	34.71 **	3.30 **	7.74 **	32.16 **	1.22	6.05 **	57.58 **	1.73 *
Alpha	7.52 **	52.66 **	3.79 **	15.64 **	27.87 **	2.94 **	19.95 **	76.79 **	3.44**
Beta	7.49 **	65.89 **	3.56 **	15.99 **	80.28 **	3.03 **	19.65 **	147.82 **	3.20**
Gamma	16.32 **	38.85 **	1.65	7.97 **	2.77	1.40	4.69 **	0.00	1.43
High	13.37 **	13.21 **	2.73 **	10.72 **	7.65 **	1.07	11.32 **	20.38 **	1.25
vHigh	7.75 **	0.78	4.90 **	9.52 **	7.41 **	2.55 **	4.09 **	8.10 **	3.95**

*There is a significant difference with *α* = 0.05.

**There is a significant difference with *α* = 0.01.

The actual classification rates for each frequency band are shown in Fig. 4 (panel C), top row (for the entire experiment). These are higher than 80% for low frequencies in the majority of pairwise comparisons of genetic strains. Further to this point, we believe that the higher discriminative power of lower frequency bands is related to the greater role these lower frequency bands play, both in the differences between behavioural phenotypes of the strains and in their response to the pharmacological agents applied. Lower frequency bands have long been implicated in ADHD in humans^67–69^, resulting in Food and Drug Administration approval of the theta/beta ratio as an additional criterion for ADHD diagnosis. The discriminative power of low frequencies further extends over network characteristic^64^ and has also been observed in the very low frequency range of EEG^70^. Further, some evidence of increased low frequency in one of the specific models of rats (SHR) used in our experiments has also been reported^71^.

**Figure 5:**
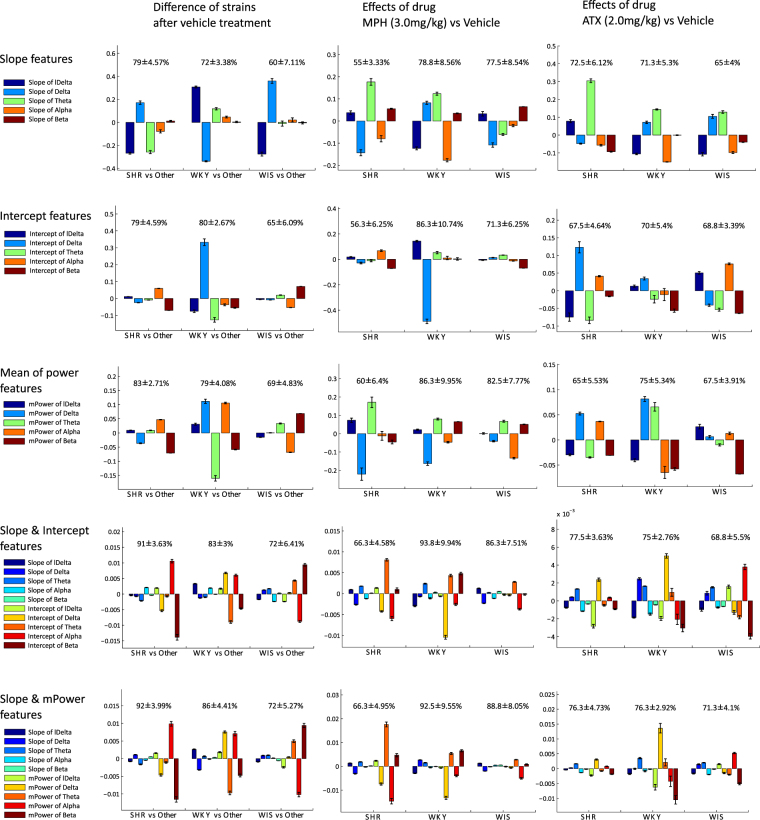
Results of average and SEM of weighting values of individual biomarkers and their classification rates. We focused on the difference in strains after the vehicle administration (left column), effects of MPH (centre column) and ATX (right column) compared with the vehicle, and discerned the weighting parameters via FDA for slope features (panel A), intercept features (panel B), mPower features (panel C), both slope and intercept features (panel D), and both slope and mPower features (panel E) in lDelta–Beta frequency bands. Bar plots depict the average and SEM of individual normalized weighting parameters, and values show average ±SEMs of classification rates via 10-fold CV test. Since the scale of slope and intercept parameters, and individual frequency bands are quite different (e.g., lDelta > delta > Theta > Alpha > Beta), individual weighting parameters were normalized depending on the individual scale, as ascertained for this visualization. Note that this normalization does not have any effect on the classification accuracy.

In the second step, we obtained the weighting parameters for the candidate frequency bands selected through FDA. Figure 5 shows the weighting parameters of the selected candidate frequency bands (lDelta–Beta), where the absolute values of the weighting parameters define the importance of the corresponding frequency bands. Finally, we consolidated the most important frequency bands selected and combined them in parsimonious functional forms as composite biomarkers according to the weighting parameters. This final step was performed heuristically. The composite biomarkers we discovered in this study, based on Fig. 5, are listed in Table 2.

**Table 2:** Biomarkers and effects on mPower.

	mPower	mPower&Slope	*λ*
Biomarker of SHR	$\frac{{\alpha }^{(p)}}{{\delta }^{(p)}{\beta }^{(p)}}$	$\frac{{\alpha }^{(p)}}{{\delta }^{(p)}{\beta }^{(p)}}+\lambda ({\delta }^{(s)}-{m}^{(s)}-{\theta }^{(s)})$	0.01
Biomarker of WKY	$\frac{{\delta }^{(p)}{\alpha }^{(p)}}{{\theta }^{(p)}{\beta }^{(p)}}$	$\frac{{\delta }^{(p)}{\alpha }^{(p)}}{{\theta }^{(p)}{\beta }^{(p)}}+\lambda ({m}^{(s)}-{\delta }^{(s)})$	0.1
Biomarker of WIS	$\frac{{\theta }^{(p)}{\beta }^{(p)}}{{m}^{(p)}{\alpha }^{(p)}}$	$\frac{{\theta }^{(p)}{\beta }^{(p)}}{{m}^{(p)}{\alpha }^{(p)}}+\lambda ({\delta }^{(s)}+{\theta }^{(s)}-{m}^{(s)})$	0.1
Effect of MPH on SHR	$\frac{{\theta }^{(p)}}{{\delta }^{(p)}{\alpha }^{(p)}}$	$\frac{{\theta }^{(p)}}{{\delta }^{(p)}{\alpha }^{(p)}}+\lambda ({m}^{(s)}+{\theta }^{(s)}-{\delta }^{(s)})$	0.01
Effect of MPH on WKY	$\frac{{\theta }^{(p)}{\beta }^{(p)}}{{\delta }^{(p)}{\alpha }^{(p)}}$	$\frac{{\theta }^{(p)}{\beta }^{(p)}}{{\delta }^{(p)}{\alpha }^{(p)}}+\lambda ({\delta }^{(s)}-{m}^{(s)})$	0.01
Effect of MPH on WIS	$\frac{{\theta }^{(p)}}{{\alpha }^{(p)}}$	$\frac{{\theta }^{(p)}}{{\alpha }^{(p)}}+\lambda ({m}^{(s)}-{\delta }^{(s)})$	0.1
Effect of ATX on SHR	$\frac{{\delta }^{(p)}}{{m}^{(p)}{\beta }^{(p)}}$	$\frac{{\delta }^{(p)}}{{m}^{(p)}{\beta }^{(p)}}+\lambda ({\theta }^{(s)}-{\alpha }^{(s)}-{m}^{(s)})$	0.01
Effect of ATX on WKY	$\frac{{\delta }^{(p)}}{{m}^{(p)}{\beta }^{(p)}}$	$\frac{{\delta }^{(p)}}{{m}^{(p)}{\beta }^{(p)}}+\lambda ({\theta }^{(s)}-{\alpha }^{(s)}-{m}^{(s)})$	0.01
Effect of ATX on WIS	$\frac{{\alpha }^{(p)}}{{\theta }^{(p)}{\beta }^{(p)}}$	$\frac{{\alpha }^{(p)}}{{\theta }^{(p)}{\beta }^{(p)}}+\lambda ({\theta }^{(s)}-{\alpha }^{(s)}-{m}^{(s)})$	0.01

## Results

The process of biomarker discovery starts with an overview of parameters computed from recorded EEG. Table 1 and Fig. 3, respectively, show the results of the ANOVA and t-test. These results allow us to evaluate the significance of multiple individual parameters computed for each strain, in this figure via their f-values, averages and standard errors of the mean (SEMs). Each individual parameter can be used to discriminate between various groupings, such as those based on strain or common dose and medication. Using individual features as markers is the first and simplest approach. Next we show that further gains in discriminative power can be obtained by combining features.

Features can be combined by linear combination. Well-established methods for this are, for example, principal component analysis (PCA) and FDA. Figure 4(A) shows the samples projected into a two-dimensional feature space via PCA and FDA from the 144-dimensional real-valued original feature space. The left PCA panel shows samples in space spanned by principal components. The variance of WIS was the smallest, the variance of SHR was the largest, and the variance of WKY was at an intermediate level. The same features projected into space spanned by the FDA components are shown in the right panel of Fig. 4(A). The panel shows three categories–genetic strains–separated into three somewhat overlapping clusters. Outliers detected by the functional boxplot were included with the samples projected in this figure; in total 150 samples are shown. Only a half of the available samples were used for inferring the FDA components to prevent over-fitting. Separation of samples into SHR and the other strains was done along the X-axis, with the vertical line (zero intercept) providing the best cluster threshold. Similarly, the horizontal line provided the threshold to separate samples into WKY and WIS clusters. FDA weights were obtained as a 144-dimensional vector. The vector was next transformed into two (8 × 9) matrices, shown in Fig. 4(B). The upper and right side bars show sums of the absolute values for each horizontal or vertical direction. Figure 4(C) shows the rounded off and averaged recognition rate matrices of Fisher’s discriminant classifier computed with the 10-fold CV test.

Exploratory experiments in the classification of strains were conducted using various combinations of features: i) slope and individual parameters for each frequency band and each experimental condition (2 dimensions), ii) both parameters of one sub-band and all experiment days (16 dimensions), iii) both parameters of one experiment day and all frequency bands (18 dimensions), iv) both parameters of all experiment days and all frequency bands (144 dimensions), and v) those of only mPower. We considered three classification objectives, for differentiation between: SHR vs WKY, SHR vs WIS, and WKY vs WIS. As a general observation, except for the ‘ALL’ sub-bands, which obviously contained all the available information, lower frequency sub-bands (i.e., lDelta–Beta) were more effective for these classification objectives than the higher frequency sub-bands (see Fig. 4(C)). SHR vs WKY and SHR vs WIS were easier to distinguish than WKY and WIS. In the cases of SHR vs WKY and SHR vs WIS, the distinction was clearer for Total-Beta bands and D0-D3 experiment days, resulting in relatively higher accuracy than for other combinations of classification entries (except for the entries labelled ALL, which contained all the information available).

The exploratory experiments were concluded by computing weights for features comprising power, slope and intercept parameters computed for selected lower frequency EEG bands. Figure 5 shows the results of the comparison using individual lower frequency sub-band-based biomarkers. There, the relative importance (weighting value) of individual biomarkers was compared using signs and lengths of bars, together with the corresponding classification performance for each objective and for each experimental condition. Furthermore, several composite biomarkers can be designed.

**Figure 6:**
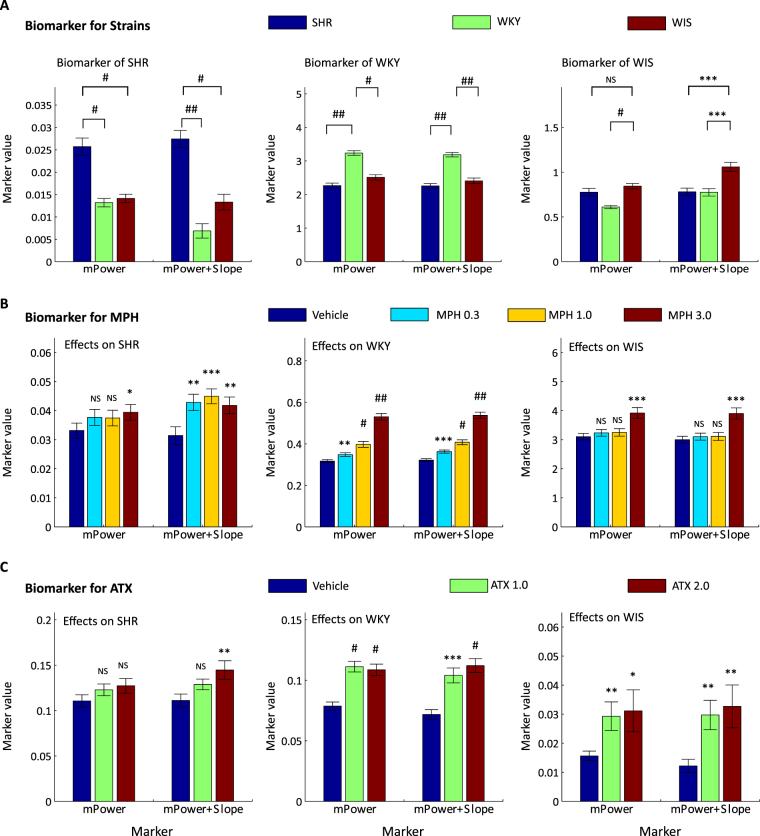
Biomarkers to identify SHR, WKY, and WIS (panel A), and to identify the effects of MPH (panel B) and ATX (panel C): ratios of mPower parameters and combinations of mPower and slope parameters are used for the construction of biomarkers. Individual bars show the averages and SEM of values of: (left in each graph) the biomarker using only mPower; and (right in each graph) the biomarker using both mPower and slope for SHR, WKY, and WIS (in left, centre and right column, respectively). In addition, significant levels (P-values) are described above the bar obtained by one-tailed t-test: *(*P* ≤ 0.005), **(*P* ≤ 0.001), ***(*P* ≤ 0.001), ^#^(*P* ≤ 10^–5^), and ^##^(*P* ≤ 10^−10^).

The final consolidation step consisted of a careful review of Figs 3–5, and translation of observed lawful relationships into parsimonious functional forms. Table 2 presents the formulas obtained for the dominant composite biomarkers using several frequency sub-bands of mPower and slope parameters. Figure 6 shows the results of values of the composite biomarkers for the determination of each rodent genetic strain and the effects of MPH and ATX. The respective biomarkers were the simplified and slightly modified versions based on Fig. 5. For example, positive alpha, negative delta, and negative beta of mPower could be important for SHR vs others. In this case, we heuristically considered $\frac{{\alpha }^{(p)}}{{\delta }^{(p)}{\beta }^{(p)}}$ as a biomarker for SHR, where ${\alpha }^{(p)}$ stands for values of mPower of alpha band, and other expressions are denoted in the same way. Next, we considered alternative heuristic combinations of slope parameters of several dominant frequency bands for the additional usage for mPower, since the slope parameter is sensitive to multiplication. For example, positive delta, negative lDelta, and negative Theta of slope were obtained by FDA for SHR vs others. In this case, we considered ${\delta }^{(s)}-{m}^{(s)}-{\theta }^{(s)}$ as a biomarker for SHR, where ^(*s*)^ stands for values of the slope. This was added to a biomarker of mPower with a multiplicative factor $\lambda :\frac{{\alpha }^{(p)}}{{\delta }^{(p)}{\beta }^{(p)}}+\lambda ({\delta }^{(s)}-{m}^{(s)}-{\theta }^{(s)})$ as a parameter. The parameter *γ* was chosen from two candidate values differing by one order of magnitude {0.1, 0.01}. The hybrid biomarkers discovered proved consistently useful in identifying SHR, WKY, WIS and the effects of individual agents on each strain.

## Discussion

In this section, we discuss the results obtained from a methodological point of view.

### Methodologies

In the experiment reported here, we used three genetic strains, namely SHR, WKY, and WIS, and two types of pharmacological agents/medications, namely MPH and ATX, and recorded EEG signals in accordance with the protocol. No such simultaneous investigation into the effects on EEG of MPH and ATX in SHR, WKY, and WIS has been considered in related research reported to date. Furthermore, to the best of our knowledge, no other study has made use of the slope and intercept parameters of the dynamical records of EEG spectral power, nor proposed a semi-automatic approach to the pharmacodynamic biomarker search by using FDA.

In this study, we used ANOVA and FDA for different objectives. However, the two methods are closely related with respect to taking into account the ratio of two kinds of variance: variance between classes and variance of error. Specifically, ANOVA is a method to test the significance of factors with respect to a control parameter considered by evaluating the ratio of variance between the classes and the variance of error. FDA maximizes the ratio of the variance between classes and the variance of error by tuning weights of parameters. In other words, ANOVA can evaluate the importance of each parameter and FDA can obtain the combinatorial weights of parameters to maximize the difference caused by some common factor. ANOVA has been widely used in this research field, for example in^71^, however FDA has not often been used. Introducing an effective usage of FDA for discovering dominant biomarkers semi-automatically is one of the contributions of this study to the research field considered. In treating large-volume data, such a semi-automatic approach could prove particularly useful.

From the results of ANOVA (Table 1), slope, intercept, and mPower parameters were significantly altered depending on the strains considered, the experimental conditions, and their interactions. In particular, the strain factors had a substantial effect on the lower frequency bands (lDelta–Beta). This suggests that lower-frequencies could be particularly important for the stratification of the rodent model and the pharmacological agent. The results of the t-test for different protocol stages (Fig. 3) show significant differences in slope, intercept, and mPower. Therefore, by introducing the slope parameter, we are able to identify significantly different protocol stages, which is not possible using the mPower parameter.

In the proposed biomarker search algorithm, we considered all the frequency bands as candidates for the biomarkers, evaluated all the sub-bands automatically, systematically removed the relatively unimportant sub-bands, and finally consolidated the relevant sub-bands into the heuristic parsimonious composite biomarkers. Using such a semi-automatic approach to biomarker design/determination, we were able to discover several dominant candidates of composite biomarkers in an unbiased way. Finally, it should be emphasized that the proposed approach is not only useful for EEG analysis of animal models of ADHD, but also potentially applicable to the EEG analysis of human subjects suffering from a range of diseases such as Alzheimer, depression, bipolar disorder or autism. Applications of the proposed semi-automatic approach to such EEG biomarker analysis may contribute to a wide range of research involving effects of pharmacological agents, especially in large-scale data analysis.

### Sensitivities of Biomarkers

In this study, we have discovered effective/dominant biomarkers using slope and mPower parameters. Table 2 summarises the biomarkers for each genetic strain and for the effects of the pharmacological agents used. Figure 1 shows the sensitivities of these biomarkers, specifically, of the biomarker using only mPower and the hybrid biomarker using mPower and slope.

From the results (Fig. 6), biomarkers discovered for the strains distinguish each strain well. In particular, the upper panel (Fig. 6A) shows how the strains can be identified using the biomarkers listed in Table 2. The structure of the graphs in the left, centre and right sub-panels corresponds with the mPower, and mPower plus slope biomarkers for the SHR, WKY and WIS strains, as listed in Table 2. Each of these strains is identified by the respective biomarkers, and the sub-panels have a distinct appearance for each biomarker. Therefore, the results for each biomarker are distinctly different, with significance levels, from the other strains indicated in the graphs.

These results reflect the difference in impulsivity between the rat strains reflected in the EEG^21,72,73^. It should be noted that EEG behaviours of WKY and WIS were similar and difficult to distinguish by standard means (see Fig. 4). However, they could be distinguished by using the newly discovered biomarkers (see Fig. 6). This is the main result of this study–proposing the methodology to devise good stratification measures for a number of different rodent genetic strains subject to different pharmacological agents.

Moreover, the biomarkers discovered for the effects of MPH and ATX well capture the effects with respect to the amounts of the pharmacological agents (Fig. 6B,C). In particular, it appears that the biomarkers for MPH effects on WKY closely reflect the amount of the pharmacological agent applied to this genetic strain (centre graph in panel B of Fig. 6).

The hybrid biomarker (mPower & slope) proved consistently to achieve superior results compared with the mPower biomarker. While the mPower-based biomarker did not distinguish between WIS and SHR, the hybrid biomarker distinguished WIS from the other strains well. The hybrid biomarker also retained or improved the significance level in the stratification of SHR and WKY. In evaluating the effects of MPH on SHR, the hybrid biomarker consistently achieved high significance, while only in one particular case of the highest MPH dosage did mPower significantly distinguish the effects on SHR. In evaluating the effects of MPH on WKY, both biomarkers achieved similar significance, however, the hybrid biomarker was more significant, even for the lowest dosage MPH 0.3. Similarly, superior results from the hybrid biomarker were obtained for the effects of ATX.

## Conclusions

We have proposed a novel methodology to capture pharmacodynamic effects of drugs on the EEG spectral power by using linear regression, to identify dominant discriminant components using FDA, and finally to consolidate them into effective biomarkers. We applied the methodology to rodent EEG records of SHR, WKY and WIS genetic strains, subject to different doses of MPH and ATX. The novel pharmacodynamic feature of ‘slope’ provided a stable, highly significant classification and served in the heuristic design of superior composite biomarkers. The proposed semi-automatic approach is a generic, parsimonious way to design such improved biomarkers using pharmacodynamic information. Using the proposed methodology, we succeeded in devising several robust biomarkers to stratify SHR, WKY, WIS genetic strains, and to capture the effects of MPH and ATX. The proposed methods can be applied to a wide range of pharmacological studies using EEG.

## Electronic supplementary material

Supplementary Information
