# Supplementary material for: Semi-Automated Biomarker Discovery from Pharmacodynamic Effects on EEG in ADHD Rodent Models
Source: Sci Rep. 2018 Mar 26;8:5202. doi: 10.1038/s41598-018-23450-y (PMC5980101; doi:10.1038/s41598-018-23450-y)
Supplement: Supplementary file 1 — Supplementary Information [file 41598_2018_23450_MOESM1_ESM.pdf]

Semi-Automated Biomarker Discovery from Pharmacodynamic Effects on EEG in ADHD Rodent Models

Tatsuya Yokota, Zbigniew R. Struzik, Peter Jurica, Masahito Horiuchi, Shuichi Hiroyama, Junhua Li, Yuji Takahara, Koichi Ogawa, Kohei Nishitomi, Minoru Hasegawa and Andrzej Cichocki

Supplementary Note 1. Median Filter

Since the data is so noisy (spiky) that it is difficult to see global aspects, a median filter was used for each time series. Supplementary Figure 1 shows an example of an original signal and its smoothed signal by using the median filter. Let us define $f(t)$ as the original signal and $y(t)$ as the smoothed signal. $y(t)$ is given by

$$y(t) = \text{Median}\{f(t+1), f(t+2), ..., f(t+W)\}, \quad (1)$$

where t is an index parameter of the time sample, W is a parameter of the window size, and 'Median$(\cdot)$' is a function that returns a median value for all inputs. In this analysis, we set $W = 480$ (i.e., 2 mins) and applied downsampling to all smoothed signals by intervals of 60 time samples (i.e., 1.5 s) for low computational cost.

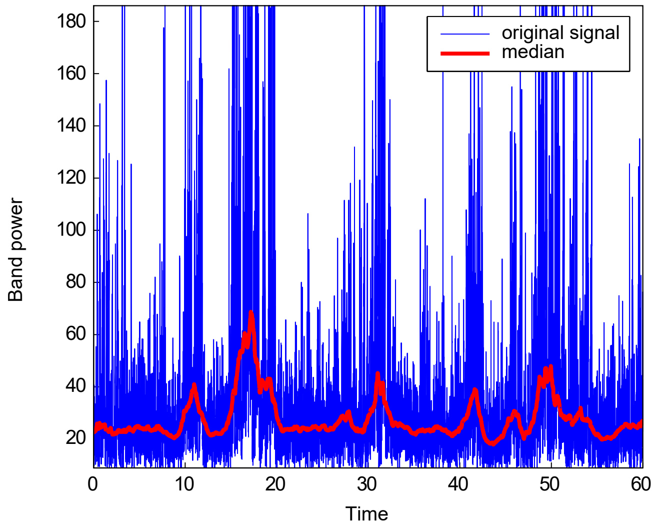

Supplementary figure 1. Smoothing by median filter: recorded EEG signals include some unavoidable noise caused by peripheral devices, sounds, motor actions and so on. Since this study focuses on comprehensive behaviour of EEG power, such noise and local spikes are removed by using the smoothing filter.

Supplementary Note 2. Outlier Detection via Functional Boxplot

After the smoothing, signals of the same type of mouse still vary. As the next step, we find a central region of the group of signals and detect outlying signals. We have a total of 216 groups, consisting of 3 types, 8 days, and 9 bands. In each group, there are 50 signals, consisting of 5 channels and 10 subjects in each type and each band. In order to find the central region of a group, we need to calculate a modified band depth (MBD) for each signal. The calculation algorithm for the MBD was proposed in [1]. The MBD of a signal is defined as an expectation value of the probability by which the signal is sandwiched between two randomly selected signals from the population of the group. In other words, the values of MBD for outside signals are small and the values of MBD for inside signals are large. After calculating the MBD, the central region is defined as being between the max and min values of signals of the 25th (i.e., 50%) largest MBD as

$$C = \{(t, c(t)) : \min_{r=1,...,25} y_r(t) \leq c(t) \leq \max_{r=1,...,25} y_r(t)\}, \tag{2}$$

where $r \in \{1, ..., 50\}$ is an index of rank of the largest MBD, and $y_r(t)$ is a signal of rth largest MBD.

To detect outliers, we estimate an inlying region as

$$\begin{aligned} C_{in} = \{(t, c(t)) : & y_{low}(t) - \lambda |y_{high}(t) - y_{low}(t)| \\ & \leq c(t) \leq y_{high}(t) + \lambda |y_{high}(t) - y_{low}(t)|\}, \end{aligned} \tag{3}$$

where $y_{low}(t) := \min_{r=1,...,25} y_r(t)$ is the lower bound of the central region, $y_{high}(t) := \max_{r=1,...,25} y_r(t)$ is the upper bound of the central region, and λ is a trade-off parameter (e.g., $\lambda = 1.5$). Outliers are signals which do not contain within the inlying region at least 100ρ % of time samples, where ρ is also a trade-off parameter (e.g., $\rho = 0.75$).

The functional boxplot shows five important curves of the deepest curve $y_1(t)$, upper bound of the central region $y_{high}(t)$, lower bound of the central region $y_{low}(t)$, inlying maximum curves $y_{\max}(t) := \max_{r \in \{\text{inlying signals}\}} y_r(t)$, inlying minimum curves $y_{\min} := \min_{r \in \{\text{inlying signals}\}} y_r(t)$, and outlying signals.

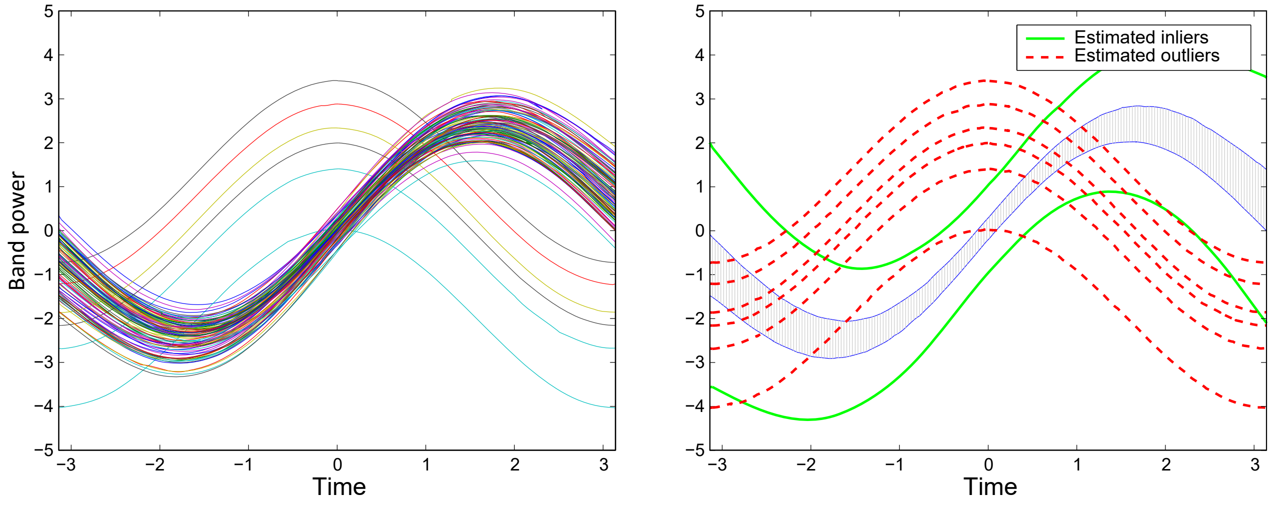

Supplementary figure 2. Example of outlier detection: (a) shows a group of 100 signals. Individual signals are illustrated by different colours. In (b), the grey area between two blue curves shows the central region, the two green curves show the upper bound and lower bound of the estimated inlying region, which is calculated by expanding the central region to be 1.5 times wider. Outliers are estimated to be signals outside the inlying region.

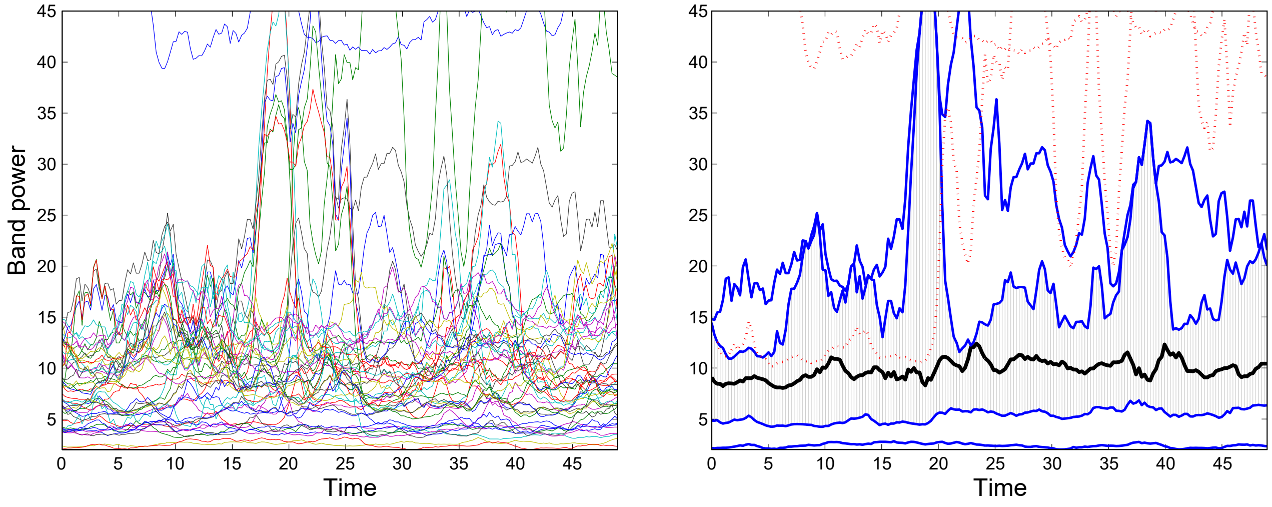

Supplementary figure 3. Result of a functional boxplot: the central black line is the deepest (median) signal, the grey area is the central region, the upper and lower blue lines are the inlying maximum and minimum curves, and the red dashed curves are the outlying signals.

References

[1] Sun, Y. & Genton, M.G. Functional boxplots. *J. Comput. Graph. Stat.* **20**, 316–334, (2011).

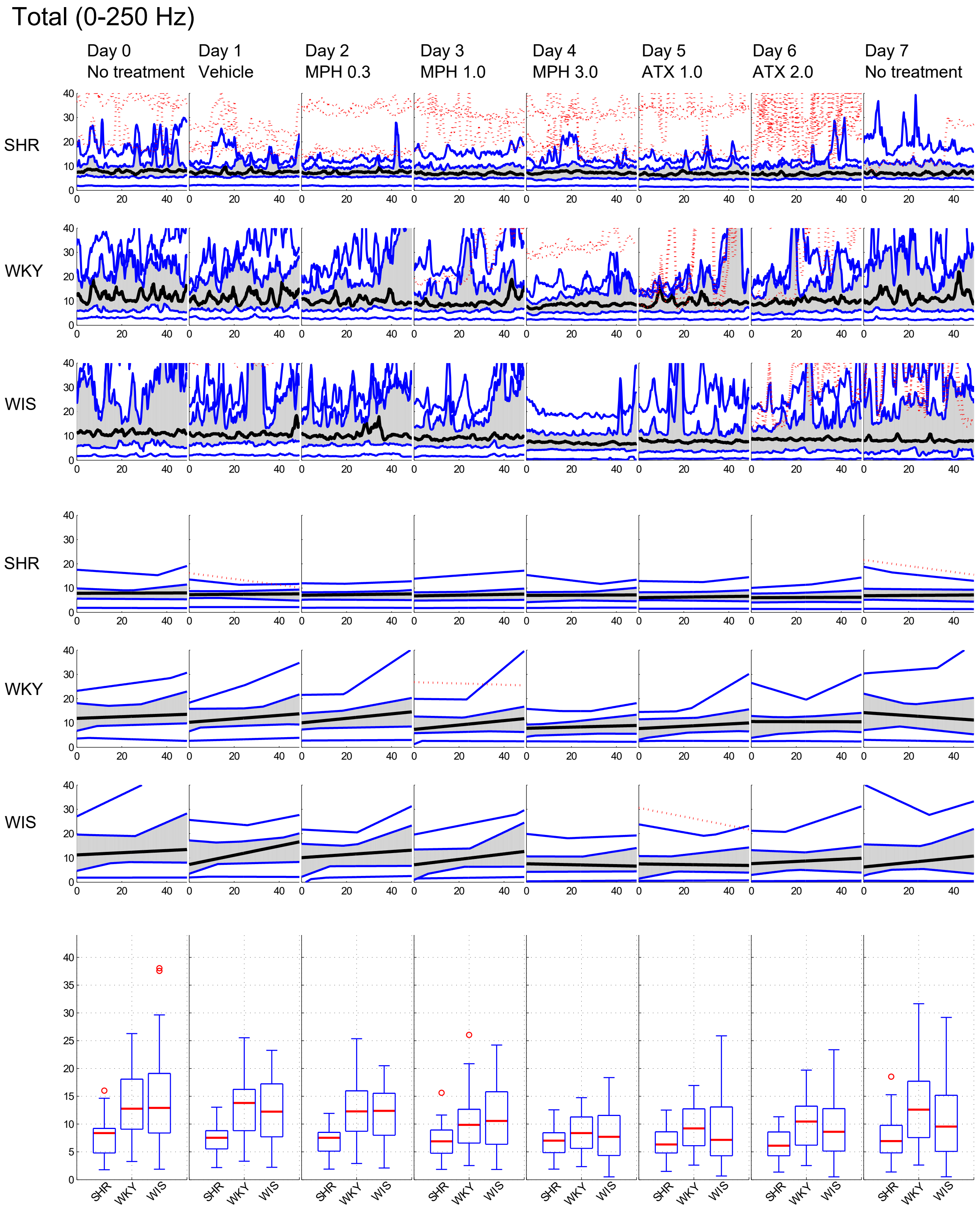

Supplementary Figure 4a. Functional boxplot of smoothed curves (top), functional boxplot of linear models (middle), and standard boxplot of mPower parameters (bottom) for the total frequency band.

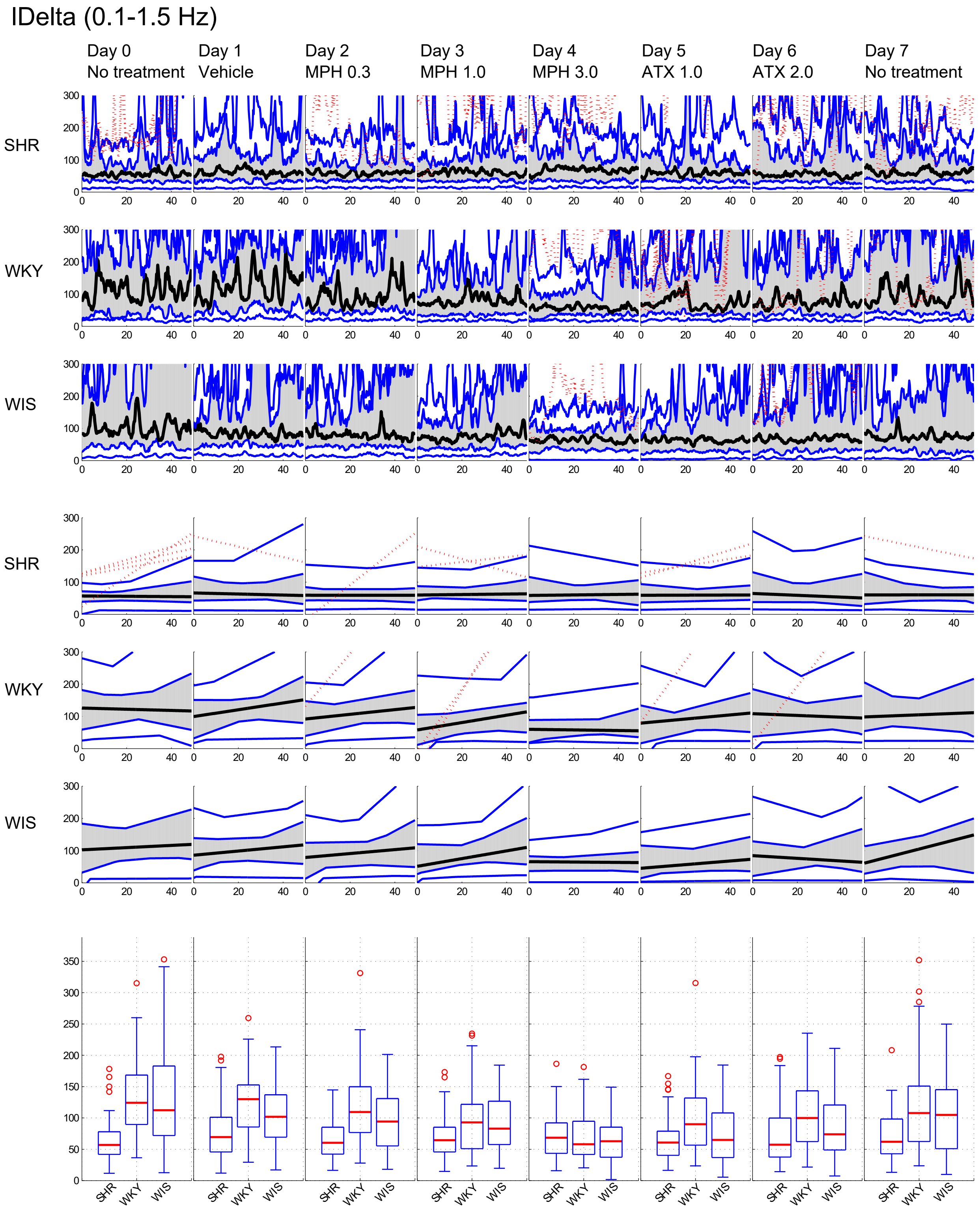

Supplementary Figure 4b. Functional boxplot of smoothed curves (top), functional boxplot of linear models (middle), and standard boxplot of mPower parameters (bottom) for the motion frequency band.

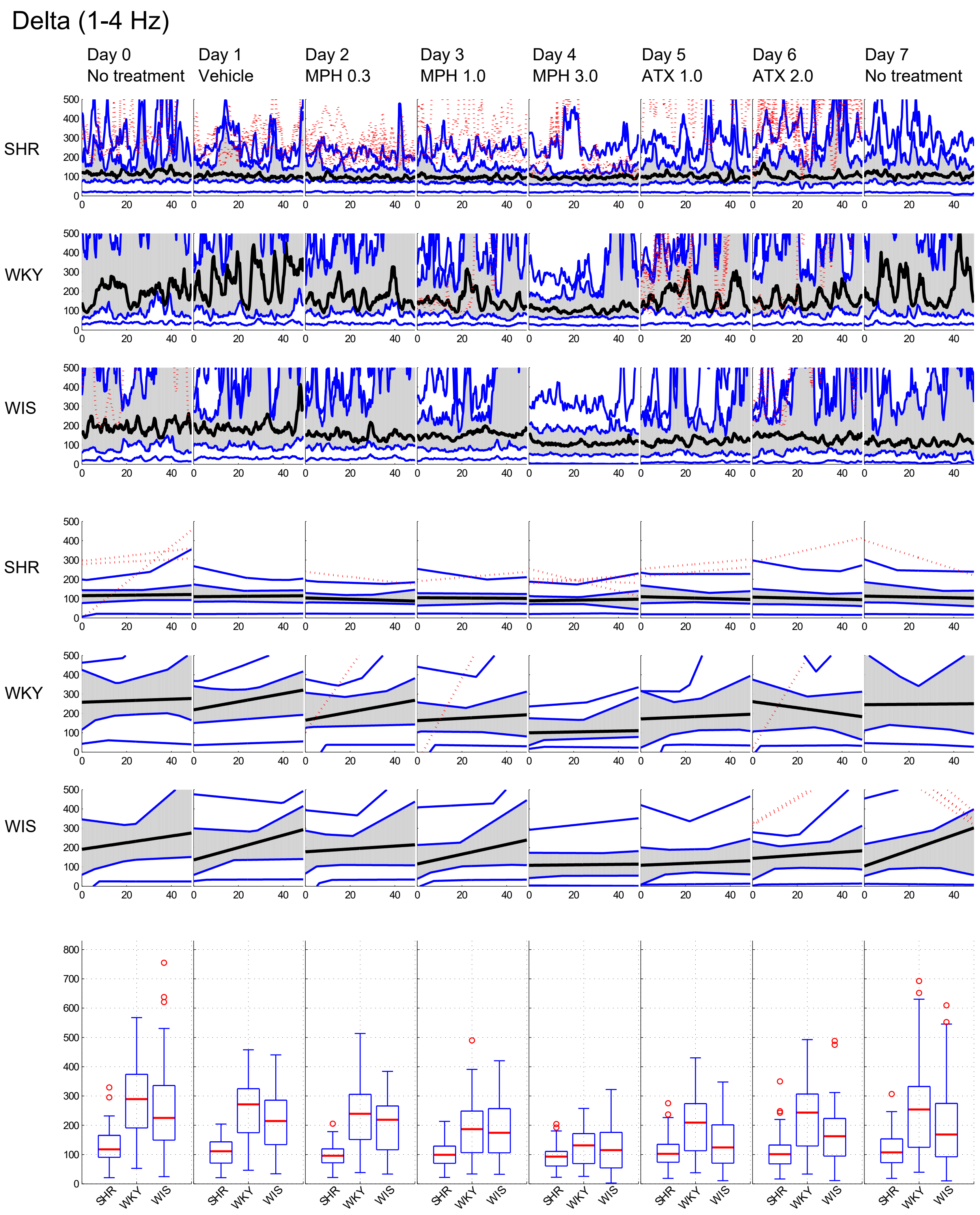

Supplementary Figure 4c. Functional boxplot of smoothed curves (top), functional boxplot of linear models (middle), and standard boxplot of mPower parameters (bottom) for the delta frequency band.

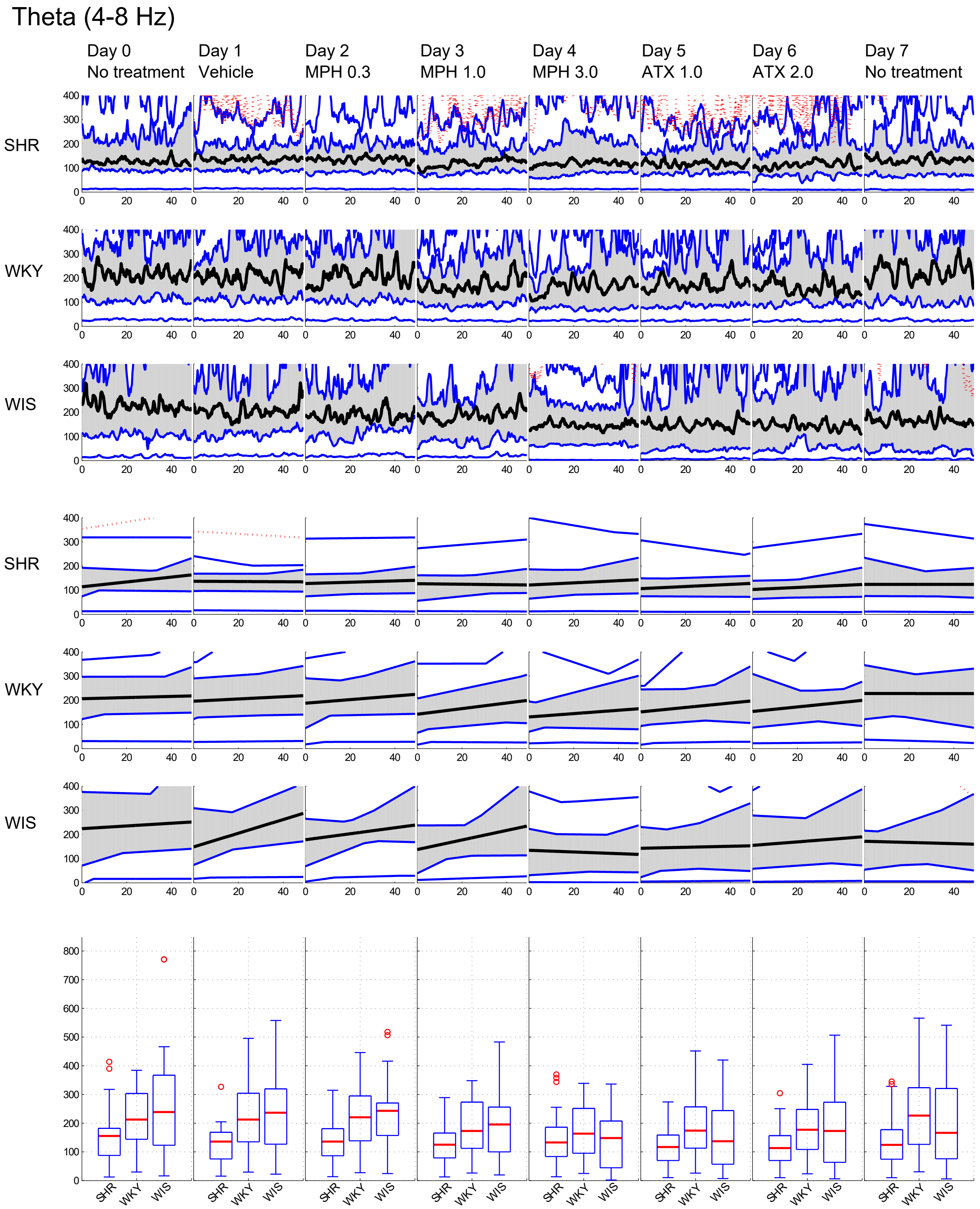

Supplementary Figure 4d. Functional boxplot of smoothed curves (top), functional boxplot of linear models (middle), and standard boxplot of mPower parameters (bottom) for the theta frequency band.

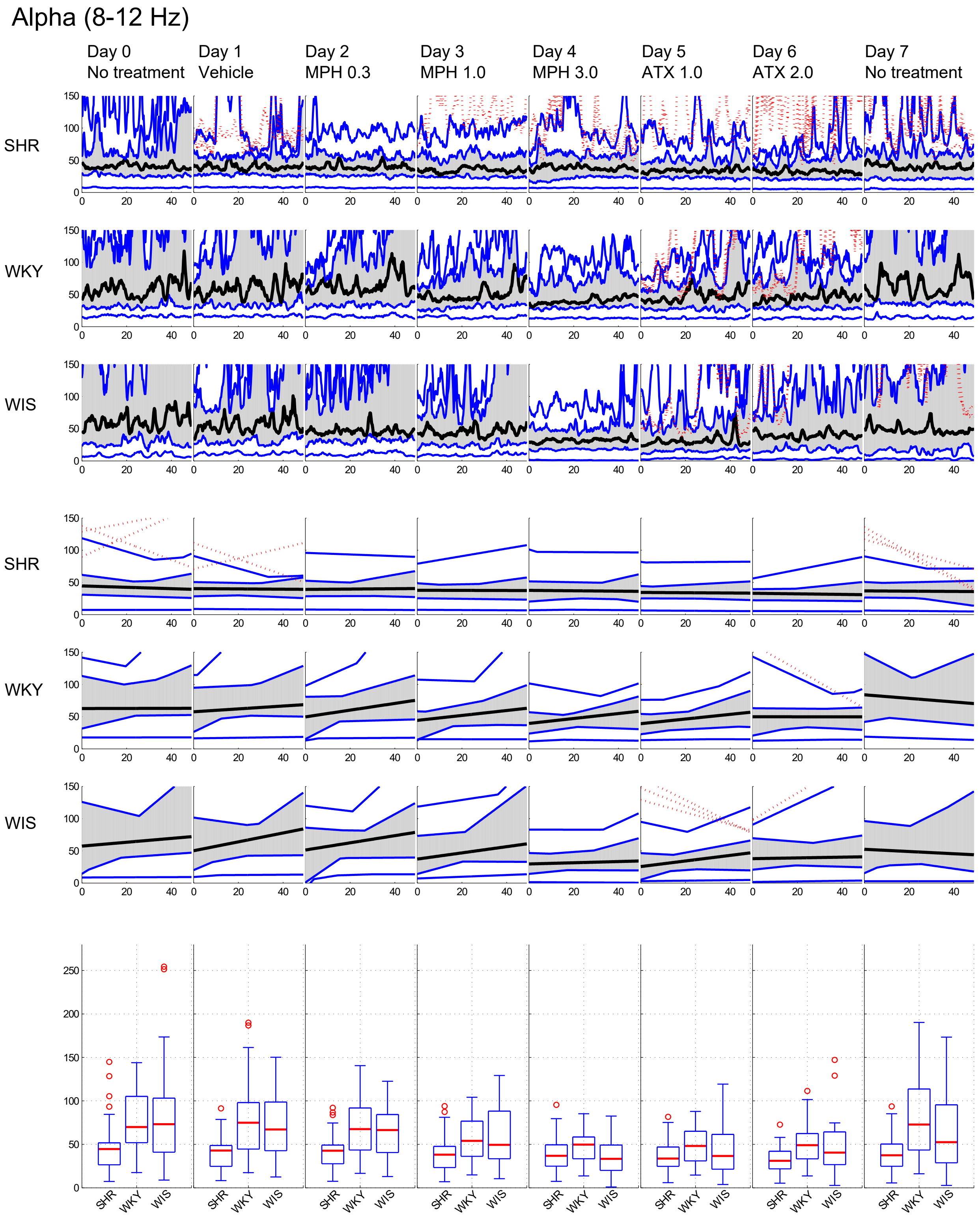

Supplementary Figure 4e. Functional boxplot of smoothed curves (top), functional boxplot of linear models (middle), and standard boxplot of mPower parameters (bottom) for the alpha frequency band.

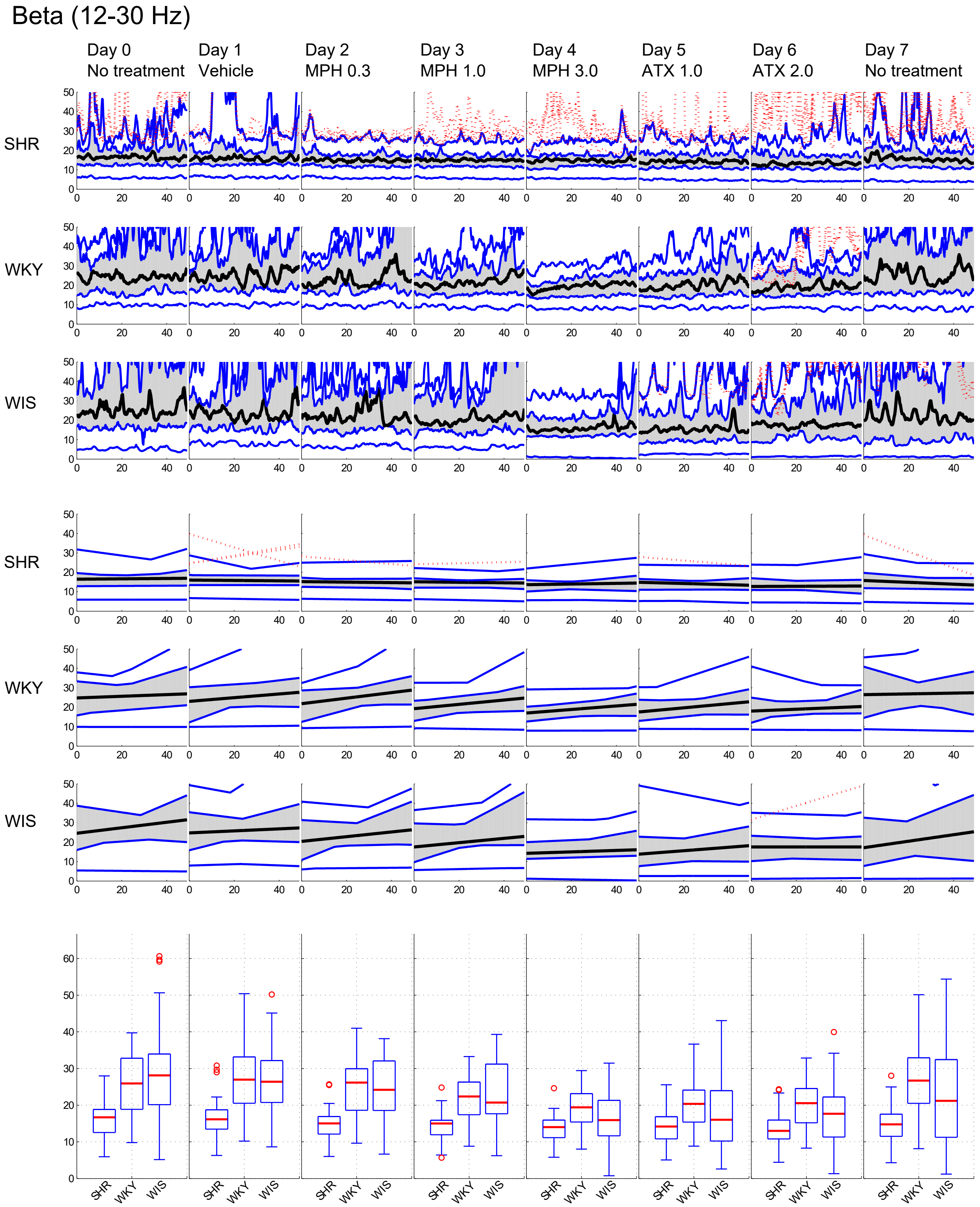

Supplementary Figure 4f. Functional boxplot of smoothed curves (top), functional boxplot of linear models (middle), and standard boxplot of mPower parameters for the beta frequency band.

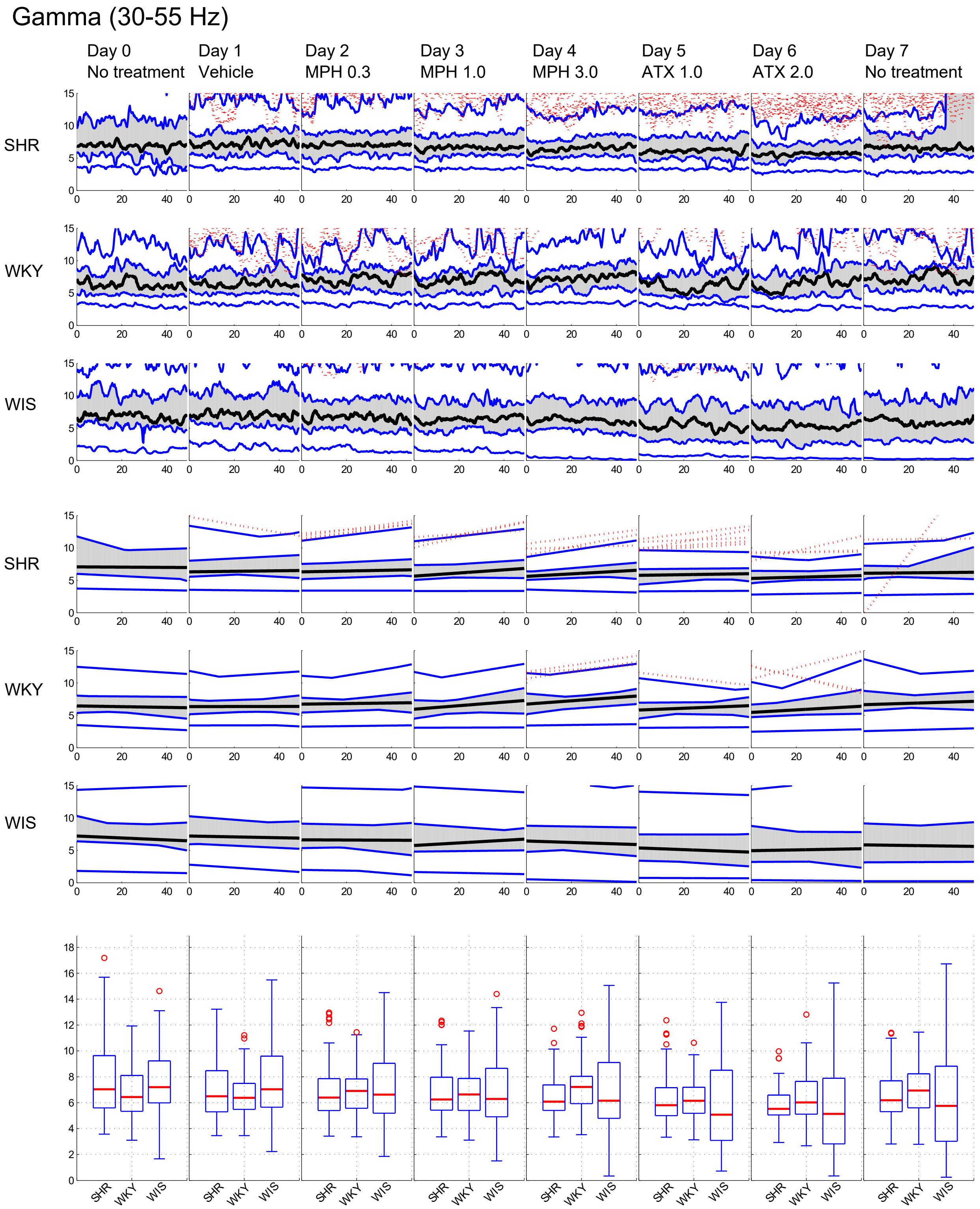

Supplementary Figure 4g. Functional boxplot of smoothed curves (top), functional boxplot of linear models (middle), and standard boxplot of mPower parameters (bottom) for the gamma frequency band.

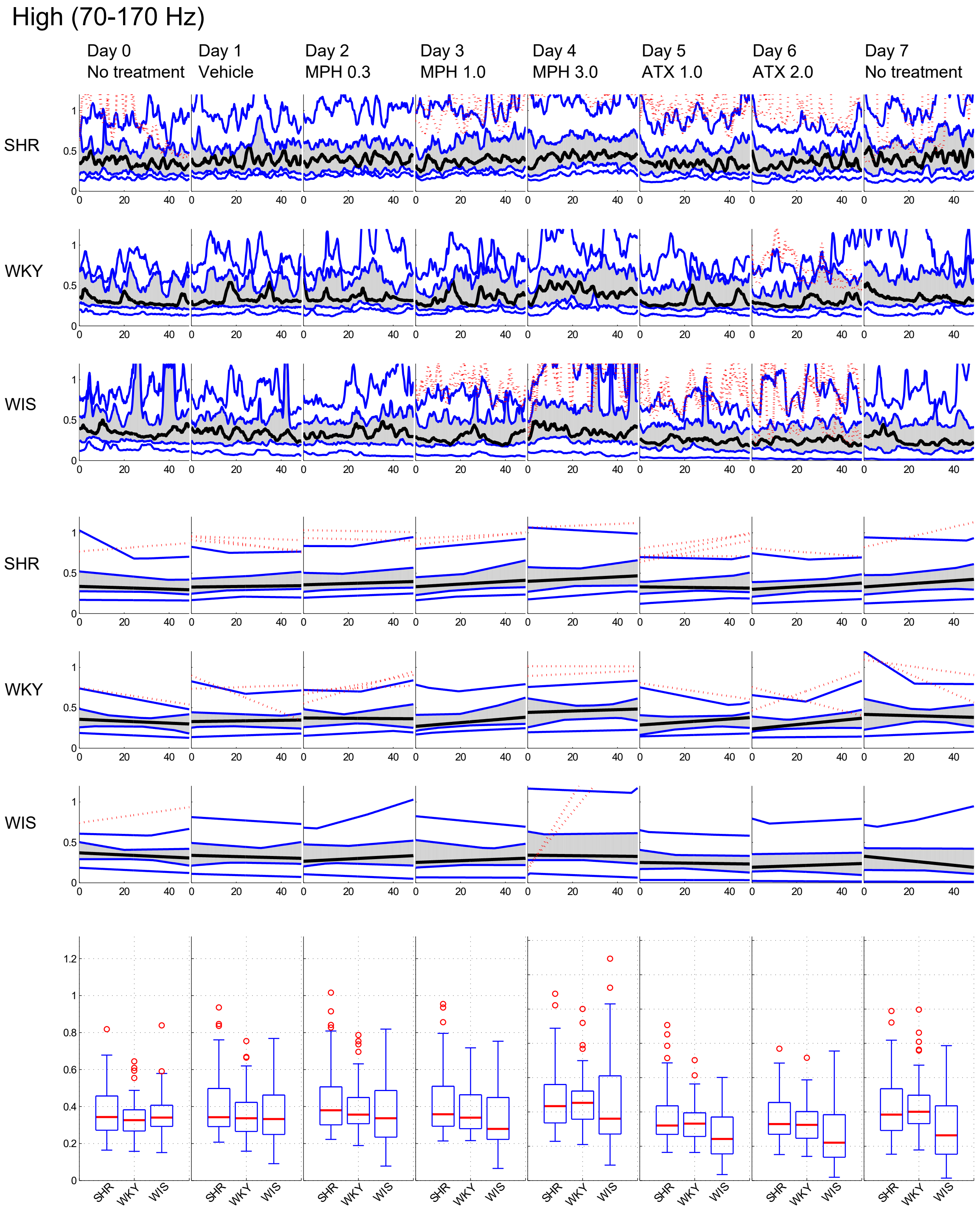

Supplementary Figure 4h. Functional boxplot of smoothed curves (top), functional boxplot of linear models (middle), and standard boxplot of mPower parameters (bottom) for the hlow frequency band.

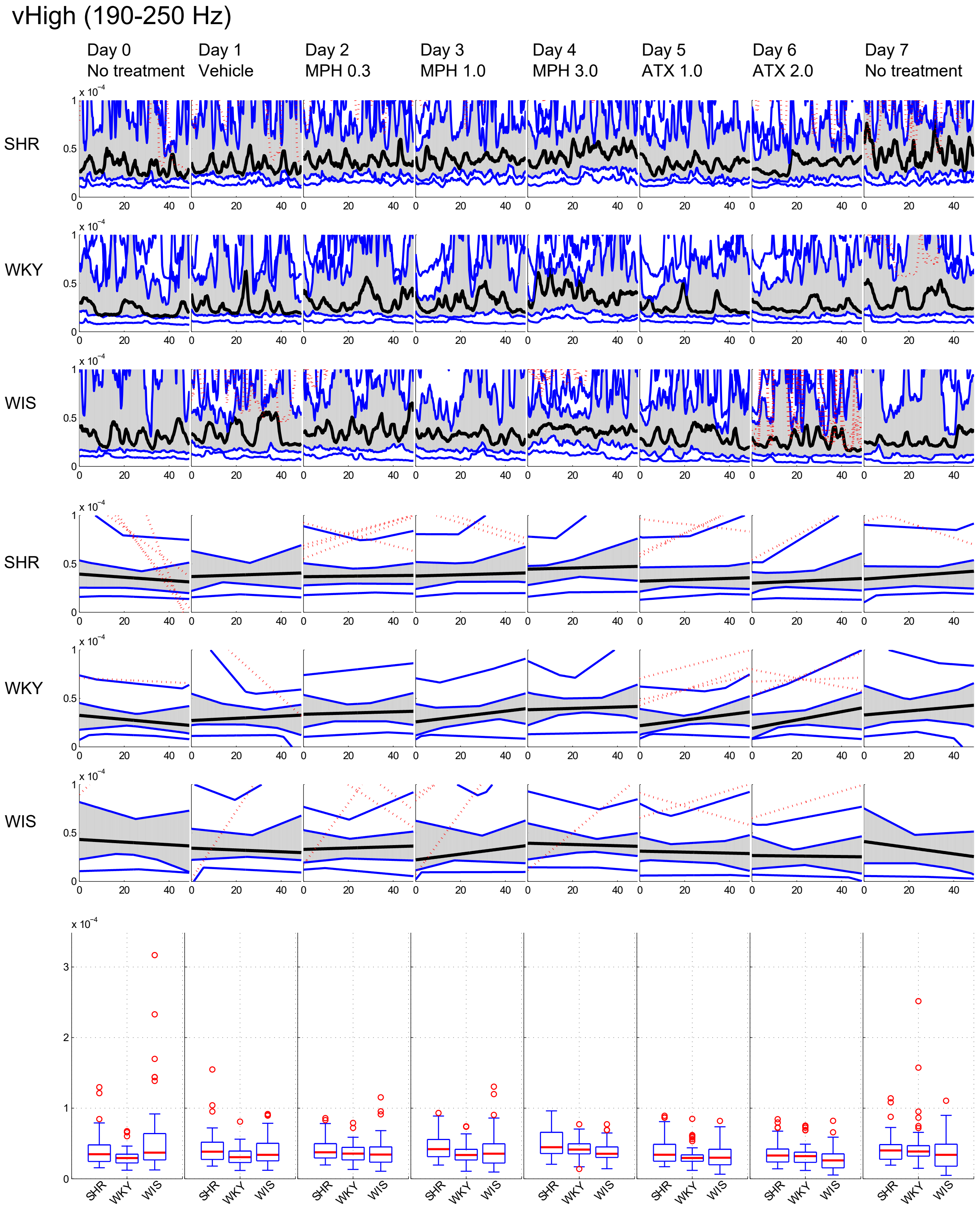

Supplementary Figure 4i. Functional boxplot of smoothed curves (top), functional boxplot of linear models (middle), and standard boxplot of mPower parameters (bottom) for the hhigh frequency band.
